# Increased Solubility and Bioavailability of Hydroxy-Cr(III) Precipitates in the Presence of Hydroxamate Siderophores

**DOI:** 10.1007/s00128-017-2234-z

**Published:** 2017-12-06

**Authors:** William E. Dubbin, Tee Boon Goh

**Affiliations:** 1 Department of Earth Science, The Natural History Museum, London, SW7 5BD UK; 2 Department of Soil Science, University of Manitoba, Winnipeg, MB R3T 2N2 Canada

**Keywords:** Chromium, Hydroxamate siderophore, Montmorillonite, Dissolution

## Abstract

Siderophores are a diverse group of low molecular weight biogenic metallophores with a particular affinity for Fe(III) but they also have potential to complex a number of other polyvalent metal cations, including Cr(III). Here we show that two hydroxamate siderophores, desferrioxamine B and rhodotorulic acid, at environmentally relevant concentrations, facilitate the dissolution of hydroxy-Cr(III) precipitates from a common layer silicate. Desferrioxamine B and rhodotorulic acid induced maximum initial Cr dissolution rates of 11.3 ± 1.7 × 10^− 4^  and 9.03 ± 0.68 × 10^− 4^ µmol m^− 2^ h^− 1^, respectively, yielding maximum solution Cr concentrations of 0.26 ± 0.01 and 0.20 ± 0.02 µmol m^− 2^, respectively. These data demonstrate that hydroxamate siderophores may play an important role increasing the dispersal of Cr in natural environments, thus facilitating greater bioavailability of this potential toxin.

Chromium occurs widely in soil and sediment and may serve as either a pollutant or an essential trace nutrient (Adriano 2001). Elevated chromium levels in soil arise through human activities such as the production of chromium waste slag from mining (Dhal et al. 2013) or they may occur naturally during the weathering of Cr-rich ultramafic rocks (Oze et al. 2007; Morrison et al. 2009). At high concentrations Cr can be toxic, posing a risk to both ecosystem health and human well-being (Adriano 2001; Guertin 2004). In Earth-surface environments Cr occurs in either the trivalent or hexavalent state, with the majority present as poorly soluble hydroxy-Cr(III) polymers sorbed to layer silicates or incorporated into Fe(III) hydroxides (Schwertmann et al. 1989; Dubbin et al. 1994; Sileo et al. 2004). Given the low solubility of hydroxy-Cr(III) precipitates at circumneutral pH Cr is generally thought not to be readily bioavailable (Bartlett and Kimble 1976; Duckworth et al. 2014).

Organic chelating ligands such as oxalate and citrate are ubiquitous in soil and they can increase the dissolution rates of sparingly soluble Cr(III) hydroxides (Rodenas et al. 1997; Carbonaro et al. 2008). The dissolved Cr(III) can then be transported to various Mn(III,IV) oxides where it is readily oxidized to the more toxic and mobile Cr(VI) (Hausladen and Fendorf 2017). Interestingly, Cr(VI) has been observed to occur naturally alongside Mn(III,IV) oxides in soils and sediments uncontaminated by anthropogenic Cr, but the mechanisms of Cr dissolution and transport in these environments are not entirely clear (Oze et al. 2007; Garnier et al. 2013). Naturally occurring low molecular weight organic ligands may well serve as key agents driving the dissolution and transport of Cr(III), thus facilitating its subsequent oxidation to Cr(VI) at the Mn oxide surface. Elucidation of the myriad pathways through which Cr is dissolved, transported and oxidised is therefore fundamental to the development of models predicting Cr cycling in natural systems.

Siderophores are a diverse group of low molecular weight biogenic chelating ligands released by plants and microbes in response to Fe stress. Although siderophores have a particular affinity for Fe(III) they also have considerable potential to complex a large number of other polyvalent metal cations, including Cr(III) (Leong and Raymond 1975; Budzikiewicz et al. 2002). Recent studies indicate that siderophores may indeed play a central role in the geochemical cycling of Cr (Duckworth et al. 2014; Stewart et al. 2016). However, despite the potential of siderophores to influence the bioavailability and fate of Cr in soils and sediments, there has to date been only limited effort to elucidate their role in the dissolution of environmentally relevant Cr-containing solids. The present work therefore examines the effect of desferrioxamine B (DFOB) and rhodotorulic acid (RA), trihydroxamate and dihydroxamate siderophores, respectively, on the dissolution of hydroxy-Cr(III) precipitates sorbed to montmorillonite. DFOB is produced by the bacterium *Streptomyces pilosus* and RA is produced by yeast of the genera *Rhodotorula*, both of which are common soil inhabitants (Fracchia et al. 2003; Manteca and Sanchez 2009). We choose montmorillonite as the sorbent because it is a common layer silicate in soils and sediments and has a high capacity to sorb hydroxy-Cr(III) polymers (Dubbin et al. 1994). Our objective was to measure, for the first time, the dissolution kinetics of Cr(III) from Cr(III)-montmorillonite in the presence of environmentally relevant concentrations of DFOB and RA. These data will better characterise the pathways through which Cr is mobilized in soils and sediments.

## Materials and Methods

Montmorillonite reacted with short-range ordered hydroxy-Cr polymers was used as the model contaminant. Briefly, 5 g Na-saturated montmorillonite (< 2 μm) (SWy-1; Clay Minerals Society) were suspended in 2 L deionized water. Predetermined quantities of CrCl_3_·6H_2_O (Aldrich) were then introduced to each of four separate montmorillonite suspensions to give four aqueous Cr(III) concentrations (67, 133, 200, 400, cmol kg^− 1^ clay) which we denote as 67, 133, 200 and 400, respectively. The four Cr(III)-montmorillonite suspensions were then titrated with 0.1 M NaOH at 1 mL min^− 1^ while stirring to achieve a NaOH/Cr^3+^ molar ratio of 2.5. The partial neutralisation of Cr^3+^_(aq)_ solutions gives rise to olation reactions and a series of hydroxy polymers, mainly dimers, trimers and tetramers as described by (Drljaca et al. 1992). The population of these oligomers increases with time while the rate of their formation varies with pH, reaching a kinetic minimum at pH 6–7, then increasing at pH > 8 (Spiccia and Marty 1986). All suspensions were diluted to 3 L, transferred to capped bottles and aged for 30 days at 23 ± 0.5°C. Following the aging period the solid was obtained by filtration through 0.02 μm pores (Millipore^®^) and the filtrate solutions were subsequently analysed for aqueous Cr by ICP-OES (Thermo iCap 6500 Duo).

The hydroxy-Cr montmorillonite reaction products were prepared as oriented mounts on sapphire substrates then characterised by X-ray diffraction (XRD) with an Enraf–Nonius PSD 120 diffractometer (Cu Kα_1_ radiation; 45 mV; 45 kV) equipped with a 120° position sensitive detector. Exchangeable Cr and cation exchange capacity (CEC) of each solid were measured by first washing the clay three times with 0.5 M CaCl_2_ to displace any loosely held Cr and to saturate the exchange sites with Ca. The excess Ca was then removed by several washings with ultrapure water (18 MΩ-cm) and the residual, bound Ca was subsequently displaced with 0.5 M MgCl_2_. The extracts were analysed for Cr and Ca by ICP-OES. The specific surface area of each Cr-montmorillonite was measured by multipoint N_2_-BET analysis (Micrometrics Gemini III 2375) after sample degassing with N_2_ at 100°C for 24 h (Brunauer et al. 1938). To ensure accuracy of measurement we analysed a reference mineral (kaolinite; 15.9 ± 0.8 m^2^ g^− 1^) alongside the Cr-montmorillonite samples.

**Fig. 1:**
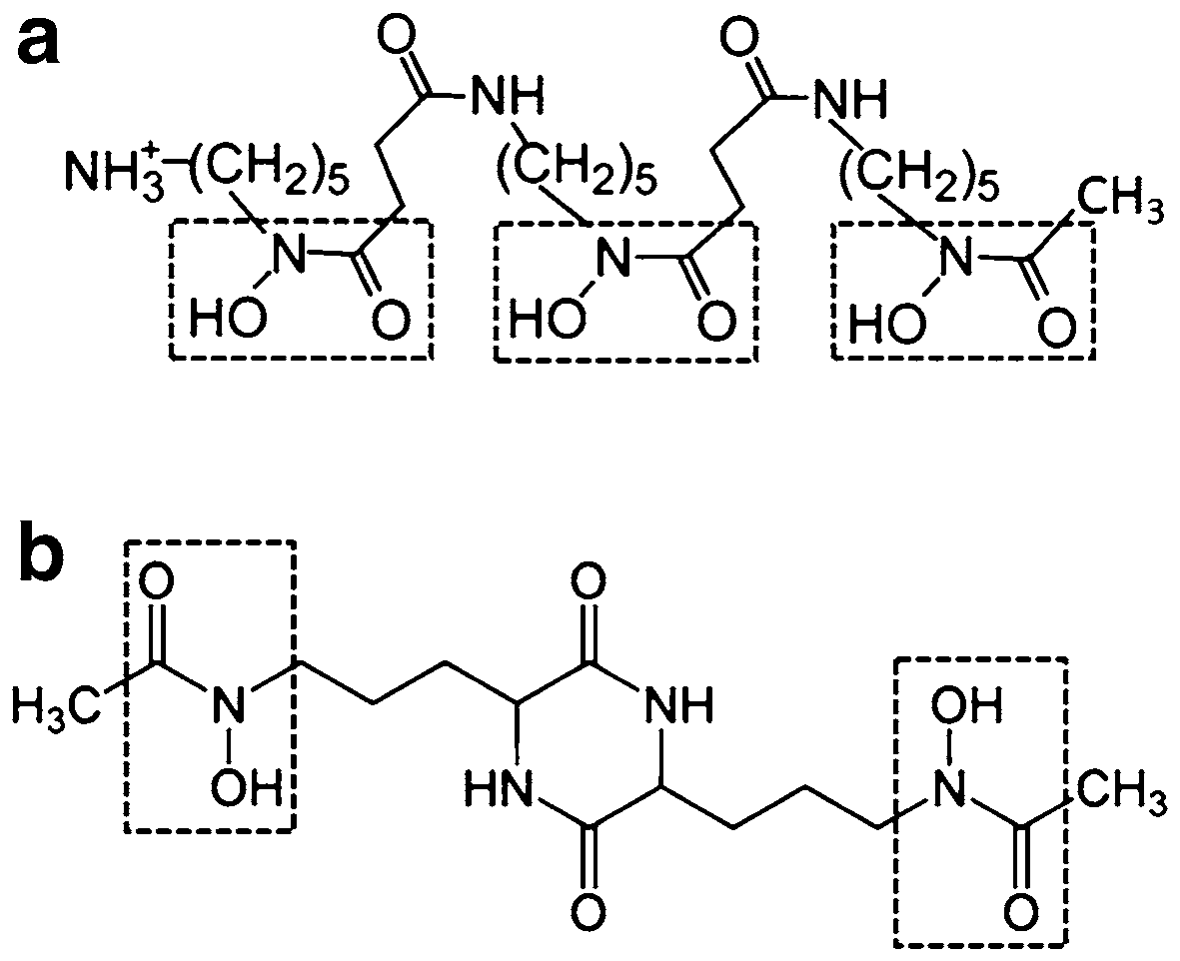
Structural representations of: **a** desferrioxamine B (DFOB) and **b** rhodotorulic acid (RA) showing the hydroxamate functional groups. The three hydroxamate groups of DFOB have p*K*_a_ values of 8.32, 8.96 and 9.55 while the two hydroxamate groups of RA give p*K*_a_ values of 8.71 and 9.88 (Martell et al. 2003)

Rates of Cr(III) dissolution from the hydroxy-Cr montmorillonite were measured in the presence and absence of the two hydroxamate siderophores (desferrioxamine B and rhodotorulic acid) (Fig. 1). To minimise proton promoted dissolution the solution pH was maintained at 6.5 throughout the duration of the reaction using a buffer solution consisting of 10 mM NaNO_3_ (BDH) and 1 mM MOPS [3-(*N*-morpholino) propanesulfonic acid; VWR]. This buffer has been used previously in comparable dissolution experiments and was found to have no significant effect on siderophore-promoted dissolution rates of metal hydroxides (Stewart et al. 2016). Portions (100 mg) of each of the four Cr(III)-montmorillonite samples were transferred to a series of 250 mL amber HDPE bottles, each containing 100 mL MOPS/NaNO_3_ buffer. A further bottle contained, as a control, 100 mg montmorillonite unreacted with Cr. To one set of montmorillonite suspensions we added a predetermined quantity of DFOB, obtained as the mesylate salt [C_25_H_46_N_5_O_8_NH_3_^+^(CH_3_SO_3_^−^)], and to a second set of montmorillonite suspensions we added RA [C_14_H_24_N_4_O_6_]. Both siderophores were obtained in crystalline form from Sigma-Aldrich and introduced to the clay suspensions as aqueous solutions. All suspensions were prepared in triplicate, brought to final volumes of 150 mL with MOPS/NaNO_3_ buffer, then left to react at 23°C on an orbital shaker (100 rpm; Orbital Incubator SI50). Each reaction vessel, except the controls, had a siderophore concentration of 120 µM.

Ten mL aliquots were removed periodically from the stirred suspensions with a syringe then filtered using a two-step process. The suspensions were first filtered through cellulose acetate filters of pore size 0.2 µm followed immediately by an additional filtration through nitrocellulose membrane filters of pore size 0.025 µm (Millipore^®^). Filtration of each 10 mL aliquot was completed within 1 min to ensure uniform sampling of all batch reactors and the obtained supernatant solutions were held at 4°C until analysis. Aqueous Cr in filtrate solutions was measured by combining 2 mL aliquots of the filtrate with 4 mL HNO_3_ (2% (v/v); SpA grade, Romil) prior to Cr analysis by ICP-MS (Agilent Technologies, ASX-7700 Series) monitoring isotope 52. To minimise polyatomic interferences from ^40^ArO^+^ and ^40^ArC^+^, the instrument was operated with 5 mL min^−1^ He (99.9995% purity) in the collision-reaction octopole cell and tuned to about 0.1% CeO/Ce. We calculate initial dissolution rates by performing least-square regression analyses. For each dissolution we chose the first five data points as these represent the most linear portion of the dissolution curve, giving regression coefficients (*R* ^2^) > 0.92 for all least square fits.

## Results and Discussion

The four prepared Cr-montmorillonite clays hold varying amounts of Cr, from 34.7 to 174 g kg^− 1^ (Table 1). Most of the added Cr was sorbed by the clay, with only the 400 clay showing significant amounts of supernatant Cr remaining at the end of the 30 day aging period. The control montmorillonite had a CEC of 91.3 cmol_c_ kg^− 1^. All four Cr-clays showed a reduction in CEC that increased with increasing amount of sorbed Cr, indicating that the hydroxy Cr polymers were bound tightly via inner-sphere complexes at the montmorillonite siloxane surface, thus blocking the exchange sites. The N_2_-BET specific surface of the control clay was 35.3 m^2^ g^− 1^ and this increased with increasing sorbed Cr such that the 400 clay gave a specific surface of 114 m^2^ g^− 1^. Given the expansion of the interlayer region as revealed by XRD (Table 1), the hydroxy Cr polymers are evidently sorbed to the internal montmorillonite surfaces, thus creating a porous framework permitting entry of the N_2_ molecule (~ 0.315 nm diameter; Lide 2004). Continued expansion of the gallery space with increasing Cr sorption further facilitates entry of N_2_ to the high surface area hydroxy Cr framework.

**Table 1:** Physicochemical properties of the montmorillonite reacted with hydroxy-Cr polymers

Supernatant Cr (cmol kg^− 1^ clay)		Cr sorbed g kg^− 1^	Basal spacing (*d*_001_) (nm)	CEC cmol_c_ kg^− 1^	Exchangeable Cr cmol_c_ kg^− 1^	Surface area^a^ m^2^ g^− 1^
Initial	Final					
0 (Control)	0	0	1.10	91.3	nd	35.3
67	0	34.7	1.36	57.2	nd	47.5
133	0	69.2	1.52	37.5	nd	87.6
200	1	103	1.73	36.0	nd	92.8
400	66	174	1.82	15.9	nd	114

*nd* Not detected within the limit of error (Cr detection limit ≈ 0.05 µg g^− 1^)

^a^Derived by multipoint N_2_-BET analysis

**Fig. 2:**
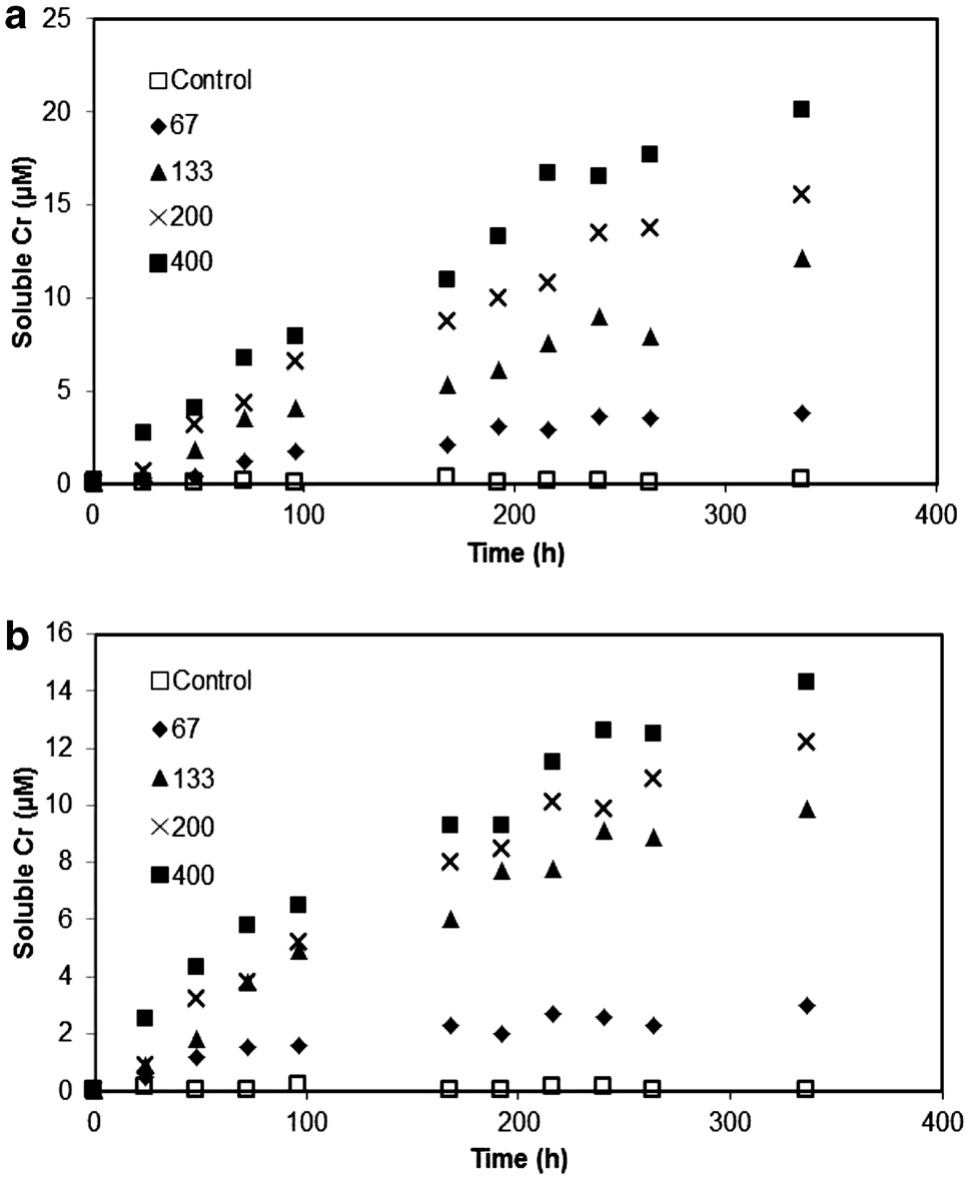
Release of Cr(III) from the four Cr(III)-treated montmorillonite clays in the presence of: **a** desferrioxamine B and **b** rhodotorulic acid to 336 h. A reference montmorillonite, untreated with Cr, serves as a control and was analysed alongside the four Cr-clays for comparison. Initial siderophore concentration: 120 µM; Cr-montmorillonite concentration: 0.67 g L^− 1^; pH 6.5; 23°C

Figure 2 shows the Cr release kinetics from montmorillonite in the presence of 120 µM DFOB and RA. In the absence of DFOB or RA there was no detectable soluble Cr over the 336 h reaction, whereas DFOB or RA presence induces significant Cr release from all four Cr-clays at all reaction times to 336 h. Maximum solution Cr concentrations increase with increasing Cr loading on the clay, and also with siderophore type, where the hexadentate DFOB yields more soluble Cr than the tetradentate RA (Table 2). These trends are most apparent for the absolute concentrations of Cr in solution, although they remain somewhat evident when normalised to surface area.

**Table 2:** Linear regression equations, surface area normalised initial dissolution rates, surface excess values for DFOB and RA, and pseudo-first-order rate coefficients for dissolution of Cr(III) from four Cr(III)-treated montmorillonite clays (67, 133, 200, 400) at pH 6.5

System	Maximum Cr in solution (µM) (µmol m^− 2^)		Regression equation	Initial dissolution rate (µmol m^− 2^ h^− 1^)	Siderophore surface excess (µmol m^− 2^)	Pseudo-first-order rate coefficient × 10^− 4^ (h^− 1^)
DFOB 67	3.8 ± 0.24	0.12 ± 0.01	y = (0.019 ± 0.002)x − 0.221 ± 0.086	5.97 ± 0.63 × 10^− 4^	1.87 ± 0.22	3.2 ± 0.3
DFOB 133	12.1 ± 1.12	0.21 ± 0.02	y = (0.047 ± 0.002)x − 0.263 ± 0.047	8.01 ± 0.34 × 10^− 4^	2.77 ± 0.25	2.9 ± 0.1
DFOB 200	15.5 ± 0.29	0.25 ± 0.03	y = (0.070 ± 0.011)x − 0.405 ± 0.316	11.3 ± 1.7 × 10^− 4^	3.92 ± 0.43	2.9 ± 0.4
DFOB 400	20.1 ± 2.61	0.26 ± 0.01	y = (0.081 ± 0.005)x + 0.435 ± 0.078	10.6 ± 0.65 × 10^− 4^	5.26 ± 0.17	2.0 ± 0.1
RA 67	3.0 ± 0.25	0.09 ± 0.01	y = (0.018 ± 0.002)x + 0.119 ± 0.051	5.66 ± 0.63 × 10^− 4^	2.67 ± 0.35	2.1 ± 0.2
RA 133	9.9 ± 0.62	0.17 ± 0.03	y = (0.053 ± 0.004)x − 0.263 ± 0.021	9.03 ± 0.68 × 10^− 4^	4.31 ± 0.29	2.1 ± 0.2
RA 200	12.2 ± 11.5	0.20 ± 0.02	y = (0.055 ± 0.009)x − 0.043 ± 0.049	8.85 ± 1.4 × 10^− 4^	7.12 ± 1.21	1.2 ± 0.2
RA 400	14.3 ± 0.39	0.19 ± 0.05	y = (0.068 ± 0.004)x + 0.556 ± 0.028	8.90 ± 0.52 × 10^− 4^	8.55 ± 0.36	1.0 ± 0.1

Initial dissolution rate was derived by dividing the slope of the regression equation by the Cr-montmorillonite concentration and surface area

Siderophore surface excess was calculated by dividing the siderophore lost from solution by the surface area of Cr-montmorillonite

Initial siderophore concentration = 120 µM

Errors represent 95% confidence interval

Cr-montmorillonite concentration = 0.67 g L^− 1^

y = soluble Cr (µM); x = time (h)

**Fig. 3:**
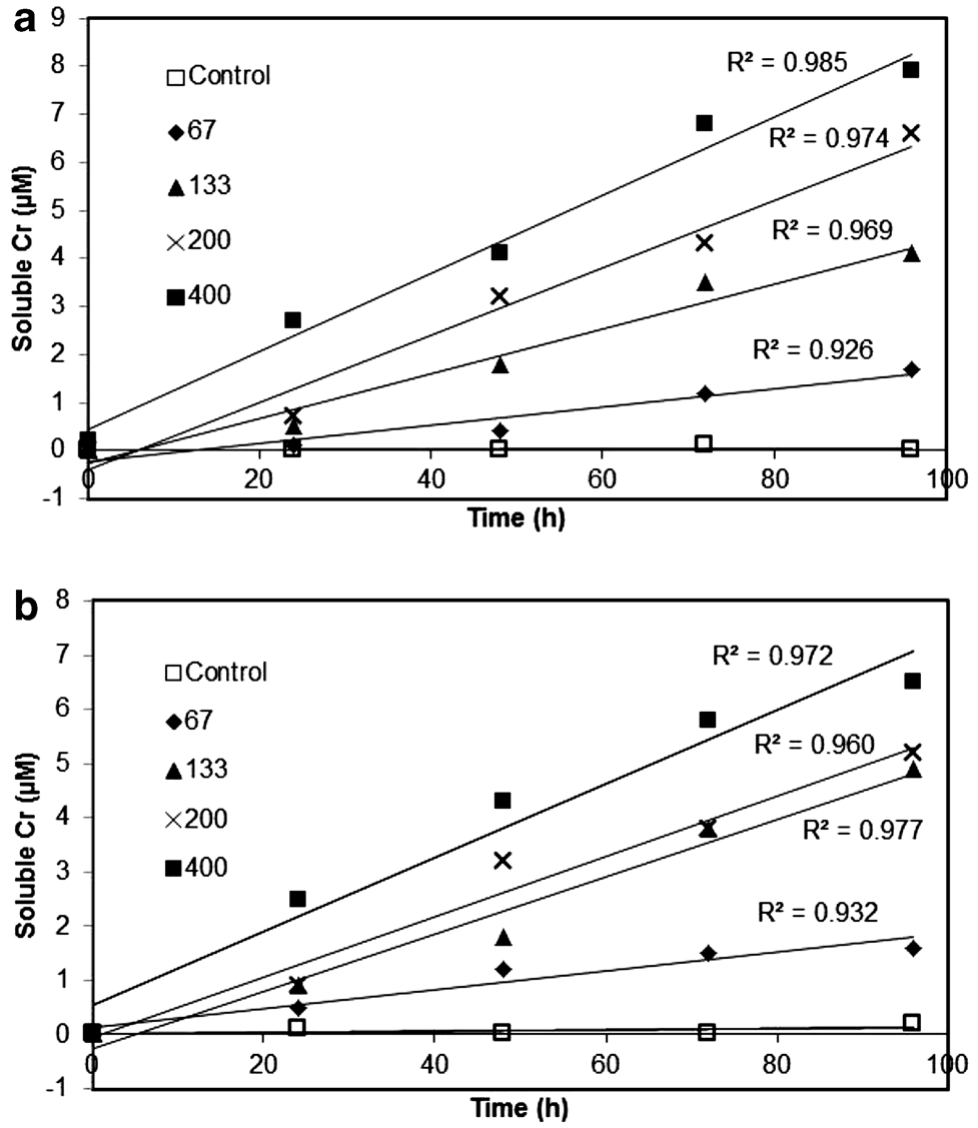
Initial dissolution kinetics showing release of Cr(III) from the four Cr(III)-treated montmorillonite clays in the presence of: **a** desferrioxamine B and **b** rhodotorulic acid. A reference montmorillonite, untreated with Cr, serves as a control and was analysed alongside the four Cr-clays for comparison. Initial siderophore concentration: 120 µM; Cr-montmorillonite concentration: 0.67 g L^− 1^; pH 6.5; 23°C

At reaction times less than 100 h Cr release approximates zero-order kinetics, where the solution Cr concentration depends linearly on time, which is typical when dissolution reactions are far-from-equilibrium (Fig. 3) (Sposito 1994; Lasaga 1998). The slope of the linear regression equation given in Table 2, column 4 is therefore equal to the zero-order rate coefficient, which increases with Cr loading for both DFOB (0.019–0.081) and RA (0.018–0.068). The slope of the regression line equation, normalised to surface area, therefore yields the initial dissolution rate (Table 2, column 5). In the presence of either DFOB or RA the initial dissolution rates generally increase with Cr loading. Interestingly, the effect of siderophore type becomes significant only for the 400 clay, where the trihydroxamate DFOB yields a significantly greater dissolution rate than the tetradentate RA (i.e. 10.6 ± 0.65 × 10^− 4^ vs. 8.90 ± 0.52 × 10^− 4^ µmol m^− 2^ h^− 1^). These dissolution rates compare with Cr release rates of 1.98 ± 0.08 × 10^− 5^ µmol m^− 2^ h^− 1^ for Cr-goethite at pH 6.5 in the presence of 270 µm DFOB (Stewart et al. 2016) and 1.4 ± 0.2 × 10^− 3^ µmol m^− 2^ h^− 1^ for Cr(OH)_3(s)_ at pH 7 in the presence of 100 mM DFOB (Duckworth et al. 2014).

Siderophore surface excess generally increases with clay surface area and Cr loading, with RA showing greater surface adsorption than DFOB at all Cr loadings (Table 2). For example, the surface excess for RA varies from 2.67 ± 0.35 to 8.55 ± 0.36 µmol m^− 2^, while that for DFOB varies from 1.87 ± 0.22 to 5.26 ± 0.17 µmol m^− 2^. For comparison, these values are approximately ten-fold greater than the surface excess for DFOB on Cr-treated goethite (0.39–0.94 µmol m^− 2^) at pH 6.5 and an initial DFOB concentration of 270 µM (Stewart et al. 2016). Given the similarly high p*K*a values for RA and DFOB (Fig. 1), both siderophores occur as cations in solution at pH 6.5 and will show little electrostatic affinity for the Cr(OH)_3_ precipitates whose p*K*a is estimated to be approximately 8.5 (Kosemulski 2009). However, the release of Cr to solution at pH 6.5 in the presence of DFOB or RA strongly indicates inner-sphere coordination of the hydroxamate groups at the Cr(OH)_3_ surface by a mechanism similar to that described for the DFOB-lepidocrocite (γ-FeOOH) system (Borer et al. 2009). Therefore, the greater surface excess of RA that we observe may be due in part to its smaller size, allowing access to interlamellar pores too small to admit the larger DFOB molecule.

Far-from-equilibrium ligand-promoted dissolution kinetics may be characterised by a pseudo-first-order rate coefficient that is obtained by dividing the surface normalised initial dissolution rate by the surface excess of the siderophore promoting the dissolution as described by Stewart et al. (2016) (Table 2). This rate coefficient therefore gives a measure of the surface excess-normalised efficacy of DFOB or RA for each Cr-montmorillonite system. The pseudo-first-order rate coefficients we derive are presented in Table 2 and decrease for each siderophore with increasing Cr loading, whilst we also observe that DFOB gives a significantly greater rate coefficient than RA for each system. Thus, the DFOB400 system gives the same rate coefficient as that for RA67 within the margin of error. These trends in rate coefficient are due mainly to an increase in siderophore surface excess that is not matched by a corresponding increase in the initial dissolution rate. DFOB and RA are evidently more effective ligands when present at lower surface coverages but this observation may also partly reflect the greater proportion of highly reactive Cr, such as monomers, dimers and trimers, at lower Cr loading. Furthermore, at high Cr loadings the montmorillonite interlayer space is populated by more hydroxy Cr precipitates (as indicated by increasing basal spacings) whose presence, while increasing surface area, may also impede movement of the siderophores throughout the interlayer gallery.

The smaller pseudo-first-order rate coefficients that we observe for RA can be explained in part by comparing the molecular geometry of the two Cr(III)-siderophore complexes. The hexadentate DFOB forms a 1:1 complex with Cr(III) such that the sixfold coordination of Cr(III) is satisfied. Also, the flexible carbon backbone of DFOB facilitates formation of both *cis* and *trans* isomers, although the *cis* isomers predominate in the chromic DFOB complexes prepared by Muller and Raymond (1984). The estimated Cr(III)-DFOB 1:1 formation constant varies from *K*_Cr_^(III)^_HDFOB_^+^ = 10^30.6^ (Duckworth et al. 2014) to *K*_Cr_^(III)^_HDFOB_^+^ = 10^33.0^ (Kruft et al. 2013) for the reaction:

$${\text{C}}{{\text{r}}^{3+}}_{{{\text{(aq)}}}}+{\text{ }}{{\text{H}}_4}{\text{DFO}}{{\text{B}}^+}_{{{\text{(aq)}}}}={\text{ CrHDFO}}{{\text{B}}^+}+{\text{ }}3{{\text{H}}^+}_{{{\text{(aq)}}}}$$

In contrast, the tetradentate, dihydroxamate RA is unable to form 1:1 octahedral complexes with Cr(III) due to ligand deficiency. Therefore RA forms bimetallic complexes of the stoichiometry Cr(III)_2_RA_3_ at circumneutral pH where the Δ-*trans* isomer dominates (Muller et al. 1985).

Although conditional formation constants are not available for Cr(III)_2_RA_3_, one can predict the relative stabilities of Cr(III)-DFOB and Cr(III)_2_RA_3_ by comparing the formation constants for each ligand with Fe(III), where *K*_Fe_^(III)^_HDFOB_^+^ = 10^32.0^ (Martell et al. 2003) and (*K*_Fe2RA3_ = 10^31.1^) (Boukhalfa et al. 2000). As these values are broadly similar, we infer that the formation constants for Cr(III)-DFOB and Cr(III)_2_RA_3_ are also broadly comparable. Therefore, the lower pseudo-first-order rate coefficients observed for RA derive not from reduced stability conferred by its hydroxamate groups, but rather from the ligand deficiency of this tetradentate siderophore.

Siderophores may serve as dispersive agents in natural environments by enhancing the mobility of contaminant metals such as Cr. This enhanced mobility can be achieved through siderophore-promoted dissolution of poorly soluble Cr containing solids and the subsequent release of dissolved Cr(III)-siderophore complexes that may be transported via advection or diffusion (Mishra et al. 2010; Duckworth et al. 2014). The dissolved Cr(III) may then be oxidized at Mn oxide surfaces and subsequently translocated as Cr(VI) to groundwaters, whose naturally occurring aqueous Cr(VI) concentrations can approach 1.4 µM, exceeding the World Health Organization’s threshold for drinking water (Oze et al. 2007). By this means Cr(III) mobilized by siderophores in the uppermost, oxic region of the critical zone may ultimately contribute to the pool of Cr(VI) in anaerobic groundwaters, environments in which siderophore production has not been observed.

However, the extrapolation of data from model contaminant systems, as described in this study, to natural environments such as soils and sediments is rarely straightforward. We therefore highlight several factors that must be considered when extrapolating siderophore behaviour from model systems to natural environments. First, as the interaction of siderophores with metals is likely competitive, an abundance of Fe(III) oxides may well diminish the siderophore-mediated dissolution of Cr(III). Second, ubiquitous low molecular weight organic acids (e.g. oxalate) act synergistically with siderophores to facilitate dissolution of Fe(III) oxides (Loring et al. 2008). However, the potential role of these organic acids in enhancing the siderophore-mediated dissolution of Cr(III)-containing solids has not yet been examined. Third, the hydroxy-Cr(III) precipitates used in this study are unlikely to be faithful proxies for those occurring in natural environments, which may, for example, complex a variety of organic substances and possess Al(III) for Cr(III) substitutions. Finally, the aqueous concentration of siderophores in soil is generally low (< 10^− 7^ M) (Powell et al. 1980) and may in fact be orders of magnitude lower than that of the many low MW organic acids. Nevertheless, given their ubiquity in soils and sediments and potential to form thermodynamically stable complexes with Cr(III), hydroxamate siderophores may play an important role in dispersal of this metal in natural environments.
